# Biological behaviors and proteomics analysis of hybrid cell line EAhy926 and its parent cell line A549

**DOI:** 10.1186/1756-9966-28-16

**Published:** 2009-02-13

**Authors:** Ze Jun Lu, Ya Qiong Ren, Guo Ping Wang, Qi Song, Mei Li, Sa Sa Jiang, Tao Ning, Yong Song Guan, Jin Liang Yang, Feng Luo

**Affiliations:** 1 Cancer Center, State Key Laboratory of Biotherapy, West China Hospital of Sichuan University, Chengdu, PR China; 2 West China Maternal and Children Hospital of Sichuan University, Chengdu, PR China

## Abstract

**Background:**

It is well established that cancer cells can fuse with endothelial cells to form hybrid cells spontaneously, which facilitates cancer cells traversing the endothelial barrier to form metastases. However, up to now, little is known about the biologic characteristics of hybrid cells. Therefore, we investigate the malignant biologic behaviors and proteins expression of the hybrid cell line EAhy926 with its parent cell line A549.

**Methods:**

Cell counting and flow cytometry assay were carried out to assess cell proliferation. The number of cells attached to the extracellular matrix (Matrigel) was measured by MTT assay for the adhesion ability of cells. Transwell chambers were established for detecting the ability of cell migration and invasion. Tumor xenograft test was carried out to observe tumorigenesis of the cell lines. In addition, two-dimensional electrophoresis (2-DE) and mass spectrometry were utilized to identify differentially expressed proteins between in Eahy926 cells and in A549 cells.

**Results:**

The doubling time of EAhy926 cell and A549 cell proliferation was 25.32 h and 27.29 h, respectively (P > 0.1). Comparing the phase distribution of cell cycle of EAhy926 cells with that of A549 cells, the percentage of cells in G0/G1 phase, in S phase and in G2/M phase was (63.7% ± 2.65%) VS (60.0% ± 3.17%), (15.4% ± 1.52%) VS (13.8% ± 1.32%), and (20.9% ± 3.40%) VS (26.3% ± 3.17%), respectively (P > 0.05). For the ability of cell adhesion of EAhy926 cells and A549 cells, the value of OD in Eahy926 cells was significantly higher than that in A549 cells (0.3236 ± 0.0514 VS 0.2434 ± 0.0390, P < 0.004). We also found that the migration ability of Eahy926 cells was stronger than that of A549 cells (28.00 ± 2.65 VS 18.00 ± 1.00, P < 0.01), and that the invasion ability of Eahy926 cells was significantly weak than that of A549 cells (15.33 ± 0.58 VS 26.67 ± 2.52, P < 0.01). In the xenograft tumor model, expansive masses of classic tumor were found in the A549 cells group, while subcutaneous inflammatory focuses were found in the EAhy926 cells group. Besides, twenty-eight proteins were identified differentially expressed between in EAhy926 cells and in A549 cells by proteomics technologies.

**Conclusion:**

As for the biological behaviors, the ability of cell proliferation in Eahy926 cells was similar to that in A549 cells, but the ability in adhesion and migration of Eahy926 cells was higher. In addition, Eahy926 cells had weaker ability in invasion and could not form tumor mass. Furthermore, there were many differently expressed proteins between hybrid cell line Eahy926 cells and A549 cells, which might partly account for some of the differences between their biological behaviors at the molecular level. These results may help to understand the processes of tumor angiogenesis, invasion and metastasis, and to search for screening method for more targets for tumor therapy in future.

## Introduction

Angiogenesis plays a critical role in the growth and progression of solid tumors. Traditionally, it is regarded that tumor vascular wall is composed of only vein endothelial cells. However, this view has been being subjected to challenges recently. Several indirect and direct evidences showed that endothelial cells and tumor cells can form "mosaic" vessels [1,2]. For example, human colon cancer cells were shown to contribute a proportion of the vessel surface in tumors grown orthotopically in mice. Even aggressive melanoma cells were found to generate vascular channels independently that facilitate tumor invasion. Cancer cells could fuse with endothelial cells to form hybrid cells both in vitro and in vivo, expressing parent proteins and chromosomal markers. The occurrence of endothelial cell markers facilitated escape of immune surveillance and clearance of the host, while the produced proteases continuously degraded the vascular basement membrane [3,4]. Therefore, studies on the cancer-endothelial hybrid cells are helpful in understanding the processes of tumor angiogenesis, invasion and metastasis.

Human endothelial-like Eahy926 cell line was derived from fusion of human umbilical vein endothelial cells with human lung adenocarcinoma cell line A549 [5,6]. In this study, malignant biological behaviors of hybrid cell line Eahy926 were investigated by comparing it to its parent cell line A549, involving in their proliferation, adhesion, invasion, migration and tumorigenesis. Meantime, 28 differentially expressed proteins were identified between Eahy926 cells and A549 cells. Moreover, some biological behaviors of Eahy926 cells were elucidated at the protein level. These data provided evidences for interactions of cancer cells with endothelial cells, and were helpful in understanding the characteristics of vascular endothelial cells, and the mechanisms of cancer invasion and metastasis.

## Methods

### Cell lines, animal and reagents

Human lung adencarcinoma cells A549 and human endothelial-like cells Eahy926 were derived from the American Type Culture Collection (ATCC). Five- to six-week-old female BALB/c mice were supplied by our State Key Laboratory of Biology. Hypoxanthine, aminopterin and thymidin were purchased from Invitrogen (Carlsbad, CA, USA). Matrigel, millicell invasion chamber and Milli-Q water were obtained from Becton Dickinson (Bedford, MA, USA). Immobiline Dry-Strips (17 cm, pH 3–10 NL), immobilized pH gradient (IPG) buffer, Dry-Strip cover fluid, urea, thiourea, ammonium bicarbonate and two-dimensional sodium dodecyl sulfate/polyacrylamide gel electrophoresis standards were purchased from BioRad (Hercules, CA, USA). And dithiothreitol, trifluoroacetic acid (TFA), acrylamide, cellulose acetate nitrate (ACN), glycerol, glycine, iodoacetamide, 3-((3-cholamidopropyl)dimethylammonio)-1-propanesulfonic acid (CHAPS), bis-hydroxymethyl-oxazoline (Bis), tetramethylethylenediamine (TEMED), sodium dodecyl sulfate (SDS), tris-hydroxymethyl-aminomethane (Tris base), 3-(4,5-dimethylthiazol-2-yl)-2,5-diphenyltetrazolium bromide (MTT), dimethylsulfoxide (DMSO), bovine serum albumin (BSA) and Coomassie brilliant blue (CBB R-250) were obtained from Sigma Chemical (St. Louis, MO, USA).

### Cell culture, cell proliferation assay and cycle analysis

Eahy926 and A549 cells were cultured in RPMI1640 media (purchased from Gibco, Langley, OK, USA) containing hypoxanthine, aminopterin and thymidin (HAT), 1% penicillin-streptomycin and 10% fetal calf serum, incubated at constant 37°C in a 5% CO_2_-humidified atmosphere. Then, cells were inoculated in a 24-well plate at 10^4 ^cells per well. Cells were counted daily for 11 days to draw the growth curves of cell proliferation. Cell cycle analysis was performed on FACSCalibur flow cytometer (Elite ESP, Beckman Coulter, Fullerton, CA, USA). The cells were stained by propidium iodide (PI; BD Pharmingen, San Diego, CA, USA), the percentages of cell population in subphases of G_0_, G_1_, S or G_2_/M were calculated from histograms by using the CellQuest software (BD Sciences, San Jose, CA, USA). The procedure was repeated for three times.

### Cell adhesion, migration and invasion assays

In the cell adhesion assay, 5 × 10^4 ^cells were plated on matrigel-precoated 96-well culture plates. After 1 h of incubation, nonadherent cells were removed, and 50 μL of MTT solution (5 mg/ml) was added to each well and incubated again at 37°C for 4 h. Then 200 μL of DMSO was added to each well. The optical density (OD) values were measured at 570 nm using a multi-well scanning spectrophotometer. Transwell chambers were established for detecting the ability of cell migration and invasion. Cell migration was evaluated by Milliwell assays (6.5-mm diameter, 8-μm pore size polycarbonate membrane). In the upper chamber 1 × 10^5 ^cells in 0.2 mL of serum-free medium were placed, while in the lower chamber medium containing 25 μg/ml fibronectin was loaded. Having migrated to the lower surface of filters, the cells were stained with hematoxylin solution. After 6 h for the second incubation, five fields in each well were counted for number of cells. Three wells were examined for each condition and cell type, and the experiment was also repeated for three times. The cell invasion assay was conducted by using 100 ml/well matrigel-precoated 24-well invasion chambers, with filters coated by extracellular matrix on the upper surface. Five fields in each well were counted after incubation for 16 h.

### Assay of tumorigenicity

Fourteen of 5 to 6-week-old female BALB/c mice were divided into two groups (seven mice per group) and inoculated subcutaneously with 200 μL of Eahy926 cell and A549 cell suspension (5 × 10^7^/ml) respectively. The growth of tumor was observed regularly. After two weeks, the mass of tumor inoculated, the liver and the lungs of mice were taken, fixed in 40 g/L formaldehyde, and cut into sections. Finally, slices of these specimens were stained with regular HE method and observed under microscope.

### Two-dimensional electrophoresis

Eahy926 and A549 cells (2 × 10^7^/ml) were solubilized in 1 ml of cell lysis solution (8 M urea, 4% CHAPS, 2 mmol/L TBP, 0.2% ampholyte, traces of bromophenol blue) on 4°C for 20 min. Insoluble material was removed by centrifugation at 15000 rpm at 4°C for 30 min. Protein concentration was determined by the method of Bradford. Samples were frozen at -70°C, and thawed immediately before use. For 17 cm IPG Ready Strips, 1 mg of protein was loaded. After rehydrating for 14 h, isoelectric focusing (IEF) was carried out for 1 h at 200 V, 1 h at 500 V and 1 h at 1000 V continuously; then a gradient was applied from 1000 to 8000 for 1 h and finally at 8000 V for 8 h to reach a total of 72 KVh at 20°C. Following IEF separation, gel strips were incubated in equilibration buffer (50 mM Tris-HCl, pH 8.8, 6 M urea, 30% glycerol, 2% SDS) with 10 mg/mL DTT for 15 min, followed in equilibration buffer with 25 mg/mL iodoacetamide for 15 min. Then strips were loaded on 12.5% SDS-PAGE gels, and electrophoresised for 20 min at a constant current of 10 mA and then at 30 mA per gel until the bromophenol blue reached the bottom of the gels. Subsequently, the gels were stained with CBB R-250, and destained with 40% methanol, then with 10% acetic acid. The experiment was replicated for five times.

### Image analysis and statistical analysis of 2-DE gel

The 12 gels were scanned with the Images Scanner GS800 (BioRad) at 300 dpi resolution. Spot detection, quantification, and the analyses of 2-D protein patterns were done with the PDQuest software (version 7.2, BioRad). Then the report of quantitative differences between two gel images was generated. The t-test was performed to compare the relative volume of spots in gels. Significant spots were selected for protein identification.

### MALDI-TOF-MS/MS analysis and database search

Excised gel pieces were destained in 50 mM NH_4_HCO_3 _buffer, pH 8.8, containing 50% ACN for 1 h, and dehydrated with 100% ACN. Then, gel pieces were rehydrated in 10 μL trypsin solution (50 mM NH_4_HCO_3_, pH 8, containing 12.5 μg/mL) for 1 h. After being incubated at 37°C overnight, 0.5 μL of incubation buffer was mixed with 0.5 μL of matrix solution (α-cyano-4-hydroxycinnamic acid, 2 mg/mL in 50% ACN, and 0.5% TFA). The sample was analyzed by Q-TOF Premier Mass Spectrometer (Waters Micromass, Milford, MA, USA). Ionization was achieved using a nitrogen laser (337 nm) and acquisitions were performed in a voltage mode. Standard calibration peptide was applied to the MALDI plate as external calibration of the instrument, and internal calibration using either trypsin autolysis ions or matrix was applied post acquisition for accurate mass determination. These parent ions in the mass range from 800 to 4000 m/z were selected to produce MS/MS ion spectra by collision-induced dissociation (CID). The mass spectrometer data were acquired and processed using MassLynx 4.1 software (Waters). The PKL format files were analyzed with a licensed copy of the MASCOT 2.0 program (MatrixScience, London, UK) against Swiss-Prot protein database with a peptide tolerance of 0.5 Da. Searching parameters were set as following: enzyme, trypsin; allowance of up to one missed cleavage peptide; the peptide mass tolerance, 1.0 Da and the fragment ion mass tolerance, 0.3 Da; fixed modification parameter, carbamoylmethylation; variable modification parameters, oxidation; auto hits allowed; results format as peptide summary report. Proteins were identified on the basis of two or more peptides, the ions scores for each one exceeded the threshold, p < 0.05, which indicated identification at the 95% confidence level for those matched peptides.

### Western blot

Western blot was done as previously described. Briefly speaking, all the cells were lysed in RIPA buffer on ice and the solutin was centrifugated at 15,000 rpm for 1 h at 4°C. Proteins were separated by 12% SDS-PAGE, and transferred to polyvinylidene difluoride membranes. The membranes were blocked in 5% skimmed milk, and subsequently probed by the primary antibodies. Then the membranes were washed and incubated with secondary antibodies conjugated with horseradish peroxidase. The immunoblot was detected using an enhanced chemiluminescence (ECL) detection system (Western Lighting™, PerkinElmer Life Science, Boston, USA).

## Results

### Cell proliferation and cell cycle

**Figure 1:**
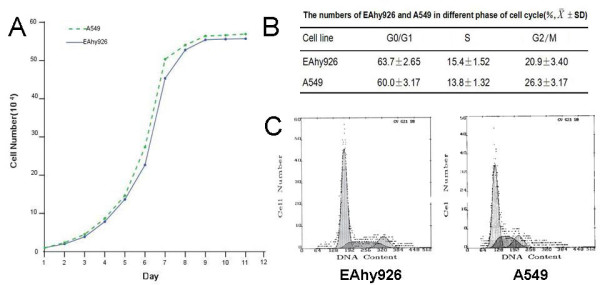
**Proliferation and cell cycle of Eahy926 and A549 cells**. (A) Cells number was counted after trypsinization every 24 hours to draw the growth curves of Eahy926 cells and A549 cells (P > 0.1); (B and C) Cell cycle analysis was performed on FACSCalibur flow cytometer. The percentages of cell population in subG_1_, G_1_, S or G_2_/M phases were calculated from histograms by using the CellQuest software; The data represent the mean ± SD of three independent experiments (P > 0.05).

MTT assay showed that the doubling time of Eahy926 and A549 cells was 25.32 h and 27.29 h, respectively (P > 0.05) (Figure 1A). Throughout the cell cycle, there was no statistical difference in each phase ratio between Eahy926 and A549 cells (P > 0.05) (Figure 1B and 1C).

### Adhesion, migration and invasion in vitro

**Figure 2:**
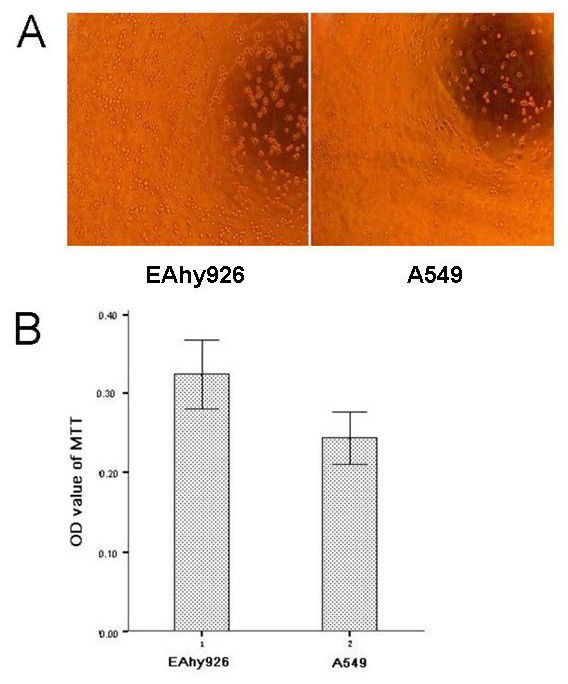
**Adhesion of Eahy926 and A549 cells with Matrigel in vitro**. (A) For adhesion test, extracellular matrix (Matrigel) was used. Representative images of Eahy926 and A549 cells adhered with the Matrigel after incubation for 1 h; (B) Number of adhesive cells with extracellular matrix (Matrigel) was measured by MTT assays. The difference in adhesion ability between Eahy926 and A549 cells was shown as OD value (OD: optical density). Independent experiments were measured in triplicate and repeated three times for each cell type; Columns, mean of independent experiments measured in triplicate and repeated for three independent times; bars, SD (P < 0.004).

**Figure 3:**
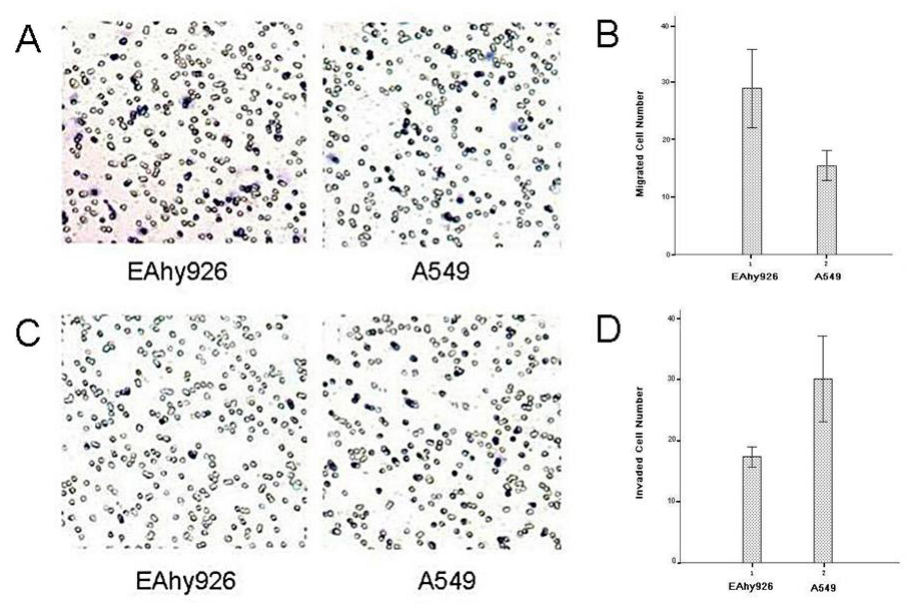
**Migration and invasion of Eahy926 and A549 cells with transwell chambers in vitro**. (A) Cell migration was evaluated by Milliwell assays. Cells migrating to the lower surface of filters were stained with hematoxylin solution. Representative images of Eahy926 and A549 cells on the lower side of a membrane after incubation for 6 h; (B) The difference in migration ability between Eahy926 and A549 cells; Columns, mean of independent experiments measured in triplicate and repeated for three independent times; bars, SD (P < 0.01); (C) Invasion assay was conducted by using invasion chambers. Representative images of Eahy926 and A549 cells on the lower side of a membrane after incubation for 16 h; (D) The difference in invasion capacity between Eahy926 and A549 cells. Columns, mean of independent experiments measured in triplicate and repeated for three independent times; bars, SD (P < 0.01).

To investigate the adhesion ability of Eahy926 and A549 cells, we counted the number of cells attached to extracellular matrix (Matrigel) by MTT assay. The adhesive ability of EAhy926 cells was found stronger than that of A549 cells. The OD value of Eahy926 cells was significant higher than that of A549 cells (0.3236 ± 0.0514 VS 0.2434 ± 0.0390, P < 0.004, Figure 2). We sequentially established Transwell chambers to detect the ability of cell migration and invasion. The migration ability of Eahy926 cells was found stronger than that of A549 cells (28.00 ± 2.65 VS 18.00 ± 1.00, P < 0.01, Figure 3A and 3B), while the invasion ability of Eahy926 cells was significantly weaker than that of A549 cells (15.33 ± 0.58 VS 26.67 ± 2.52, P < 0.01, Figure 3C and 3D).

### Tumorigenicity in vivo

**Figure 4:**
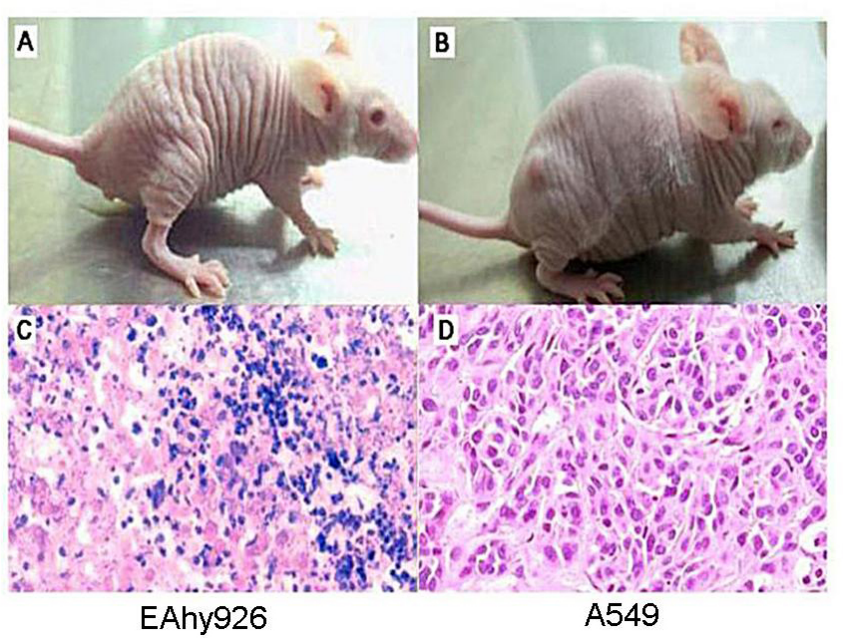
**Tumorigenicity of Eahy926 and A549 cells in vivo**. (A) No tumor mass formed roughly within 14 days after s.c. injection of Eahy926 cells; (B) Tumor mass formed roughly within 10 days after s.c. injection of A549 cells; (C) On day 14 after s.c inoculation of Eahy926 cells; tissues collected from the inoculative site were identified as inflammatory necrosis in the Eahy926 cells group; (D) On day 14 after s.c inoculation of A549 cells, classic tumor microstructure was found in the A549 cells group and the rate of tumorigenicity was 100%.

In order to test tumorigenicity of these cells, 1 × 10^6 ^Eahy926 cells or A549 cells were subcutaneously (s.c) injected into the nude mice. However, no tumor was observed in any mouse on the 14^th ^day in the Eahy926 cells group (Figure 4A). In the A549 cells group, tumors formed in each nude mouse on the 10^th ^day after the s.c. injection (Figure 4B). Tissues collected from the inoculation site were identified as inflammatory necrosis of the Eahy926 cells group, while in such tissues collected from the A549 cells group, masses of classic tumor microstructure were found (Figure 4C and 4D). Moreover, tumor invasion and metastasis to organs such as the liver and the lungs were not found by histological examination in both groups.

### Comparative proteomics analysis

**Figure 5:**
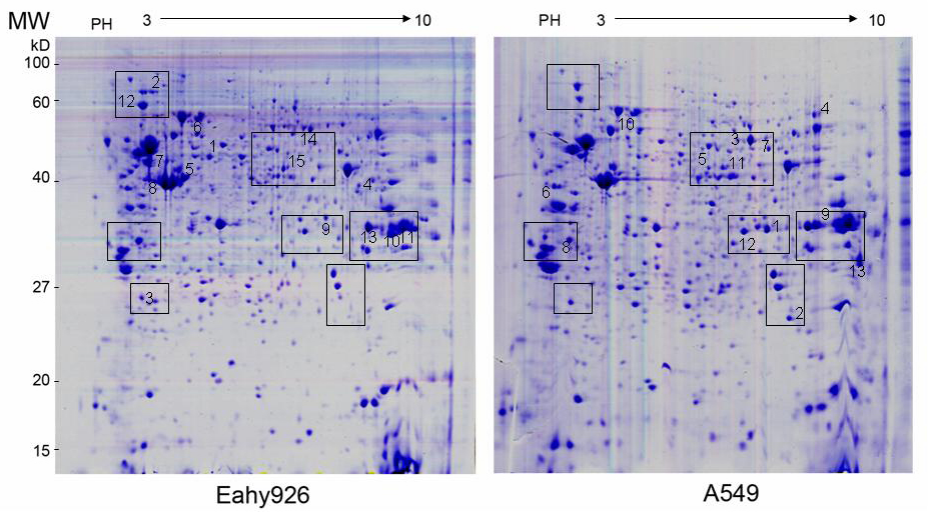
**Analysis of differentially expressed proteins by 2-DE (two-dimensional electrophoresis)**. Two-dimensional electrophoresis based proteomics approaches were performed to determine the proteins expressed differently. Representative 2-DE gels of Eahy926 and A549 cells. Differential expression protein spots were labeled with numbers.

**Figure 6:**
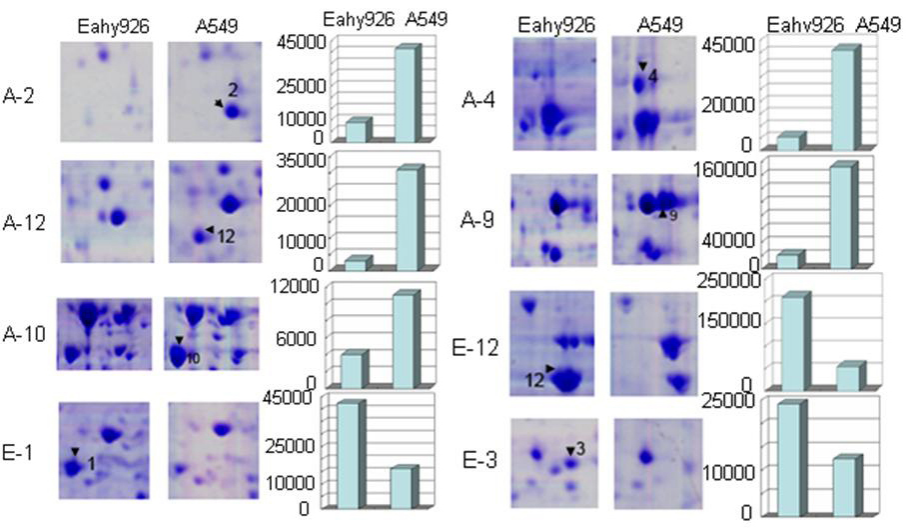
**Close-up image of partial differential expression of protein spots between Eahy926 and A549 cells**. Protein spot discrepancies were arrowed and marked with number. Each bar graph showed expression level of protein spots in Eahy926 and A549 cells.

**Figure 7:**
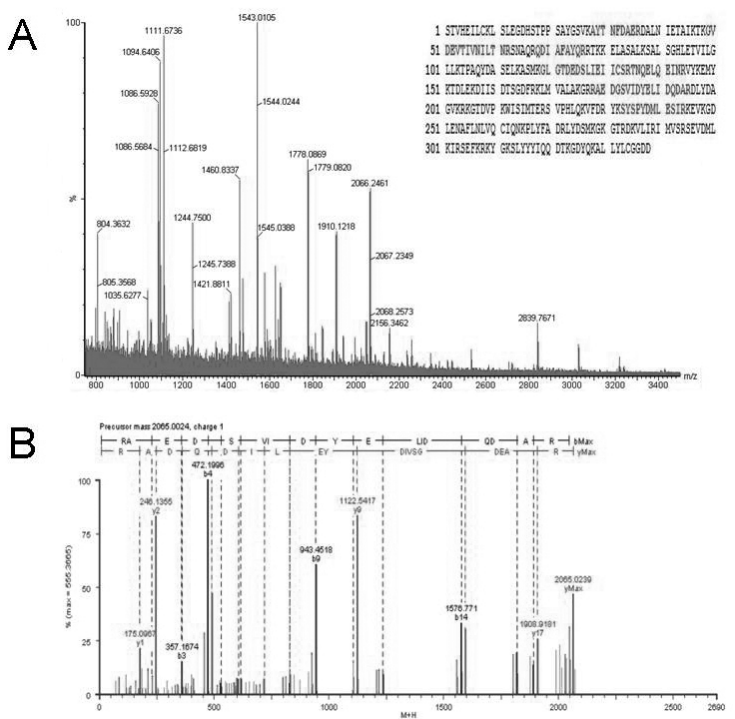
**MS spectra of tryptic peptides from spot A-9 (Annexin A2)**. (A) Peptide mass fingerprinting (PMF) of the trypsin-cleaved spot A-9. The sequence of Annexin A2 protein was represented by single-letter code for amino acids on the top right corner of the image and it was exhibited by red bold. Sequence coverage: 26%; (B) MS-MS sequence analysis of one of the parent ions, m/z value 2065.0024. The matched sequence was identified as RAEDGSVIDYELIDQDAR.

Two-dimensional electrophoresis based proteomics approach was performed to determine the differently expressed proteins. The images of 2-D gel of both Eahy926 cells and A549 cells were shown in Figure 5 and 6. Twenty-eight proteins, involved in cell proliferation, differentiation, signal transduction and so on, were identified by peptide mass fingerprinting (PMF) and tandem mass spectrometry (TMS) (Table 1). The PMF and TMS maps of Annexin A2 were presented in Figure 7. Of the 28 proteins identified above, 15 were found overexpressed in Eahy926 cells, while 13 were overexpressed in A549 cells.

**Table 1:** List of identified proteins differentially expressed between Eahy926 and A549 cells

**Spot ID**	**Swiss**^a^)	**Gene name**	**Protein name**	**Function**	**T^b^) PI**	**T^c^) *Mr***	**Score^d^)**	**Idi^e^)**	**Ex^f^) E/A**
A1	P15121	AKR1B1	Aldose reductase (AR)	metabolism	6.56	36099	50	TMS	down
A2	P04179	SOD2	Superoxide dismutase [Mn]	metabolism	8.35	24878	38	TMS	down
A3	P11413	G6PD	Glucose-6-phosphate 1-dehydrogenase	metabolism	6.44	59553	276	PMF/TMS	down
A4	P29401	TKT	Transketolase (TK)	metabolism	7.58	68519	119	PMF/TMS	down
A5	P50395	GDI2	Rab GDP dissociation inhibitor beta	metabolism	6.11	51807	164	PMF/TMS	down
A6	P06748	NPM1	Nucleophosim (NPM)	metabolism	4.64	32726	116	PMF/TMS	down
A7	P43490	NAMPT	Nicotinamide phosphoribosyltransferase	metabolism	6.69	55772	57	TMS	down
A8	P31947	YWHAQ	14-3-3 protein sigma	differation/proliferation	4.68	27871	57	TMS	down
A9	P07355	ANXA2	Annexin A2 (Annexin?)	calcium ion binding	7.56	38677	347	PMF/TMS	down
A10	P10809	HSPD1	60 kDa heat shock protein	molecular chaperone	5.70	61187	370	PMF/TMS	down
A11	O75306	NDUFS2	NADH-ubiquinone oxidoreductase	metabolism	7.21	52911	37	TMS	down
A12	P60891	PRPS1	Ribose-phosphate pyrophosphokinase?	metabolism	6.56	35194	103	PMF/TMS	down
A13	P15559	NQO1	NAD(P)H dehydrogenase	metabolism	8.91	30905	38	TMS	down
E1	P05787	KRT8	Cytokeratin-8 (CK-8)	structural	5.52	53540	131	PMF/TMS	up
E2	P08238	HSP90AA1	Heat shock protein HSP 90	molecular chaperone	4.94	84875	58	TMS	up
E3	P07858	CTSB	Cathepsin B precursor (Cathepsin B)	migration/inv-asion	5.28	38766	84	TMS	up
E4	P62333	PSMC6	26s protease regulatory subunit	metabolism	7.10	44430	76	TMS	up
E5	P05783	KRT18	Cytokeratin-18 (CK18)	structural	5.34	47897	107	PMF/TMS	up
E6	P48643	CCT5	T-complex protein (TCP-1) (CCT)	molecular chaperone	5.45	60089	82	TMS	up
E7	P08670	VIM	Vimentin	structral	5.06	53545	38	TMS	up
E8	P68032	ACTC	Alpha-cardiac action	migration/inv-asion	5.23	42334	57	TMS	up
E9	P00491	NP	Purine nucleoside phosphorylase (PNP)	metabolism	6.45	32325	64	TMS	up
E10	P00338	LDHA	L-lactate dehydrogenase A (LDH-A)	metabolism	8.46	36819	41	TMS	up
E11	P22626	HNRPA2B1	hnRNP A2/B1	differation/proliferation	8.97	37464	173	PMF/TMS	up
E12	P11021	HSPA5	78 kDa glucose-regulated protein	molecular chaperone	5.07	72402	299	PMF/TMS	up
E13	P63244	GNB2L1	Guanine nucleotide-bingding protein	signal transduction	7.56	35380	199	PMF/TMS	up
E14	P31948	STIP1	Stress-induced-phosphoprotein 1	molecular chaperone	6.40	63227	30	TMS	up
E15	P26641	EEF1G	Elongation factor 1-gamma	structural	6.27	50298	113	PMF/TMS	up

a) Swiss: SWISS-PROT accession number; b) T pI: theoretical isoelectric point of the matching protein; c) T Mr: theoretical relative molecular mass of the matching protein; d) Score: the score of PMF and TMS; e) Idi: identification method; TMS: tandem mass spectrometry; PMF: peptide mass fingerprinting; f) Ex E/A: expression level in Eahy926/A549 cells

### Western blot verification

**Figure 8:**
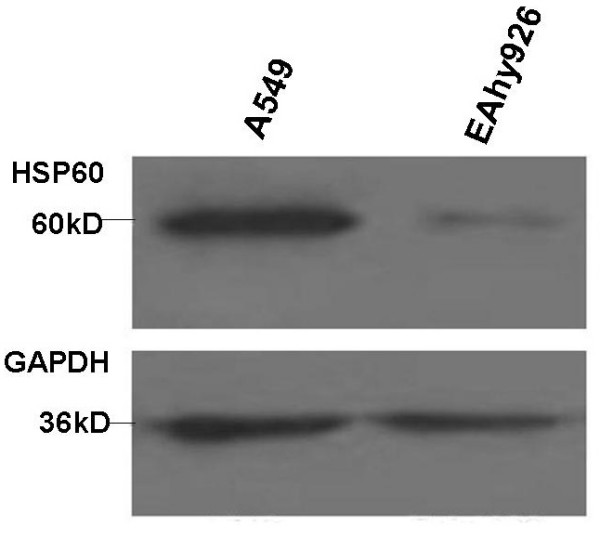
**Western blot analysis of Hsp60**. Western blot was performed to verify the expression of HSP60 in A549 and Eahy926 cells. The expression of HSP60 in A549 cells was higher than that in Eahy926 cells.

To verify the expression of HSP60 protein in both A549 and Eahy926 cells, western blot was performed. Expression of HSP60 protein was identified in both A549 cells and Eahy926 cells, and overexpression of this protein was found in the former (Figure 8). The result was consistent with the findings on 2-D gels about HSP60 protein.

## Discussion

Interactions of cancer cells with vascular endothelial cells are very complicated [7,8]. Cancer cells and endothelial cells communicate with each other and influence angiogenesis through the formation of gap junctions [9]. Moreover, cancer cells can fuse with endothelial cells to form hybrid cells spontaneously both in vivo and in vitro. The hybrid cells are viable and able to undergo mitosis. Importantly, after fusion with endothelial cells, cancer cells acquire some of the characteristics of endothelial cells temporarily or permanently, which is involved in promotion of tumor invasion and metastasis.

Human endothelial-like Eahy926 cell line was derived by fusing human umbilical vein endothelial cells with the permanent human cell line A549. Hybrid cell line Eahy926 had more chromosomes than either of its progenitor cell types had. However, there were few researches on the difference in biological behaviors and expression of proteins between the hybrid cells and its parent cells recently. Here we obtained several results regarding the difference in biological behaviors and protein expression between the hybrid cells Eahy926 and its parent cells A549. Cell counting and cycle analysis assays showed that the proliferation ability of Eahy926 cells was similar to that of A549 cells. Why did not significant difference exist for cell proliferation and cell cycle in both cell lines? The reason for this may be as following. Firstly, with fused cancer cells, hybrid cells could acquire malignant cell proliferation characteristics of cancer [3,5,10]. Secondly, the transformation of endothelial cells after fusion might cause an alteration in their receptors and signal transduction systems, which also affect their affinity for and responses to growth factors [11].

In this study, twenty-eight differentially expressed proteins, related to cell proliferation, differentiation, apoptosis, invasion and metastasis, were identified by proteomics technologies in the cell lines. At the same time, it was found that the adhesion ability with Matrigel of Eahy926 cells were stronger. In fact, the long fusiform morphology of Eahy926 cells was similar to the endothelial cells, which was associated with the higher adhesion ability. In addition, the up-regulation of cell surface adhesion molecules such as ICAM-1 and VCAM-1 also enhanced the cells adhesion [12].

In this paper, we also found that the migration of Eahy926 cells was more but the invasion was less than those of the parental cell line, and that xenograft tumor failed to form in the nude mouse. By coincidence, other researchers also found that the invasion ability of Eahy926 cells was weaker than that of HT1080 fibrosarcoma and C8161 melanoma cells, but was stronger than that of umbilical vein endothelial cells [13]. Actually, molecular biological mechanisms on this phenomenon have not been elucidated completely. Annexin A2, a Ca^2+^-binding protein, has a function in promoting tumor cells invasion and metastasis through its interaction with matrix proteins [14,15]. Annexin A2 was found down-regulated in Eahy926 cells (Table 1, Figure 6). Reduction of annexin A2 resulted in the weaker invasion and tumorigenesis ability of Eahy926 cells. CK18, CK8 and cathepsin B were involved in cell malignant transformation and the destruction of basement membranes by degrading collagen and laminin, promoting tumor migration [16-19]. These proteins were found up-regulated in Eahy926 cells (Table 1, Figure 6). Therefore, the higher migration ability of Eahy926 cells shown in this study could be accounted for partially at the protein level. However, it was difficult to explain all the biological behaviors only by the proteins founding. For instance, GRP78, as a heat shock protein, was implicated in protecting tumor cells from cytotoxic damage and apoptosis. Over-expressed GRP78 has been correlated with tumor invasion and metastasis in the xenograft nude mouse model [20-22]. Although GRP78 was up-regulated in this study, Eahy926 cells had the weaker invasion ability than A549 cells had and failed to form xenograft tumor in nude mice. There were many factors influencing the cell's biological behaviors. Several researches suggested that many hybrid cells, derived from fusion of cancer cells with normal cells, had the weaker tumorigenesis [23,24]. But, hybridoma cells used in producing monoclonal antibodies had stronger tumorigenesis. Additionally, another hybrid cell line, derived from fusion of human cervical carcinoma cells HeLa with human diploid fibroblasts, was also found to be non-tumorigenic completely in vivo [25]. The probable causes lay in transferring of the tumor suppressor gene and the different responses to the growth regulatory signals [26,27]. In the present study, we investigated malignant biological behaviors and protein expression of hybrid cell line Eahy926 comparatively. Having considered the complex formation process of hybrid cells, further study should be made to explore the complex interactions of tumor cells with endothelial cells. This would not only contribute to the elucidation of the accurate processes of tumor angiogenesis, invasion and metastasis, but also be helpful in screening more molecular targets for the development of novel therapeutic approaches.

## Conclusion

Our study suggested that the proliferation ability of Eahy926 cells was similar to that of A549 cells, but the ability in adhesion and migration of Eahy926 cells was higher. In addition, Eahy926 cells had weaker ability of invasion and could not form tumor mass. Furthermore, there were many differently expressed proteins between hybrid cell line Eahy926 cells and A549 cells, which might partly account for some of the differences between their biological behaviors at the molecular level. These results may help to understand the processes of tumor angiogenesis, invasion and metastasis, and to search for screening method for more targets for tumor therapy in future.

## Competing interests

The authors declare that they have no competing interests.

## Authors' contributions

ZJL and YQR drafted the manuscript and carried out the cell adhesion, migration and invasion assays. GPW and ML performed the 2-DE and western-blot. QS and SSJ performed the cell culture, cell proliferation assay and cycle analysis. TN performed MALDI-TOF MS studies. YSG helped in drafting and polishing the manuscript. JLY and FL participated in the design of the study. All authors read and approved the final manuscript.
